# Supplementary material for: Generalizable links between symptoms of borderline personality disorder and functional connectivity
Source: bioRxiv. 2023 Aug 21:2023.08.03.551534. Preprint. [Version 2] doi: 10.1101/2023.08.03.551534 (PMC10473667; doi:10.1101/2023.08.03.551534)
Supplement: 1 [file NIHPP2023.08.03.551534V2-supplement-1.pdf]

bioRxiv preprint doi: https://doi.org/10.1101/2023.08.03.551534; this version posted August 21, 2023. The copyright holder for this preprint (which was not certified by peer review) is the author/funder, who has granted bioRxiv a license to display the preprint in perpetuity. It is made available under aCC-BY 4.0 International license.

Table S1. NEO Five Factor Inventory (NEO-FFI) items included in BPD proxy score | The NEO-FFI was used to estimate composite BPD proxy scores (19). The included 24 items and the corresponding questions are listed below. Given that a lower score in items assessing *Agreeableness* and *Conscientiousness* corresponds to more severe BPD symptoms, these items were reverse scored (i.e., multiplied by -1). The composite BPD score was estimated for each participant as the mean score across the included 24 items.

Neo-FFI Factor	Facet	Item Number and Question
Neuroticism (N)	Anxiety	#21: “I often feel tense and jittery.”
		#31: “I rarely feel fearful or anxious.”
	Angry Hostility	#36: “I often get angry at the way people treat me.”
	Depression	#26: “Sometimes I feel completely worthless.”
		#41: “Too often when things go wrong, I get discouraged and feel like giving up.”
	Vulnerability	#11: “When I'm under a great deal of stress, sometimes I feel like I'm going to pieces.”
		#51: “I often feel helpless and want someone else to solve my problems.”
Extraversion (E)	Excitement-Seeking	#22: “I like to be where the action is.”
Openness (O)	Feelings	#33: “I seldom notice the moods or feelings that different environments produce.”
	Actions	#8: “Once I find the right way to do something, I stick to It.”
		#28: “I often try new and foreign foods.”
Agreeableness (A)	Trust	#24: “I tend to be cynical and skeptical of others' intentions.”
		#29: “I believe that most people will take advantage of you if you let them.”
	Straightforwardness	#59: “If necessary, I am willing to manipulate people to get what I want.”
	Altruism	#14: “Some people think I'm selfish and egotistical.”
		#39: “Some people think of me as cold and calculating.”
	Compliance	#9: “I often get into arguments with my family and co-workers.”
		#54: “If I don't like people, I let them know it.”
Conscientiousness (C)	Order	#5: “I keep my belongings clean and neat.”
		#55: “I never seem to be able to get organized.”
	Dutifulness	#40: “When I make a commitment, I can always be counted on to follow through.”
		#45: “Sometimes I'm not as dependable or reliable as I should be.”
	Self-Discipline	#30: “I waste a lot of time before settling down to work.”
		#50: “I am a productive person who always gets the job done.”

bioRxiv preprint doi: https://doi.org/10.1101/2023.08.03.551534; this version posted August 21, 2023. The copyright holder for this preprint (which was not certified by peer review) is the author/funder, who has granted bioRxiv a license to display the preprint in perpetuity. It is made available under aCC-BY 4.0 International license.

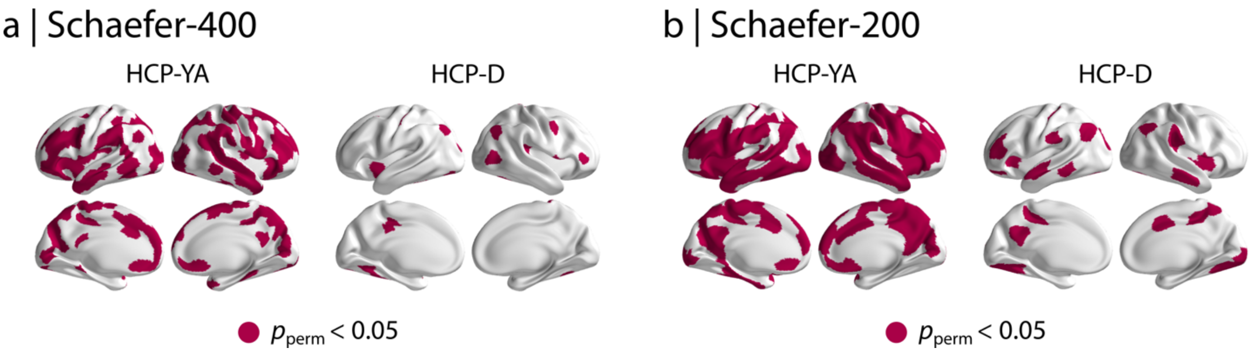

Figure S1. Regional predictive capacity thresholded based on permutation tests | We generated a null distribution of predicted scores for each regional model using 1,000 permutations by randomly shuffling the dependent variable (i.e., BPD scores) and re-calculating the model performance for each permutation. Regional maps were then thresholded based on permutation tests following correction for multiple comparisons ($p_{perm} < 0.05$; FDR-corrected).

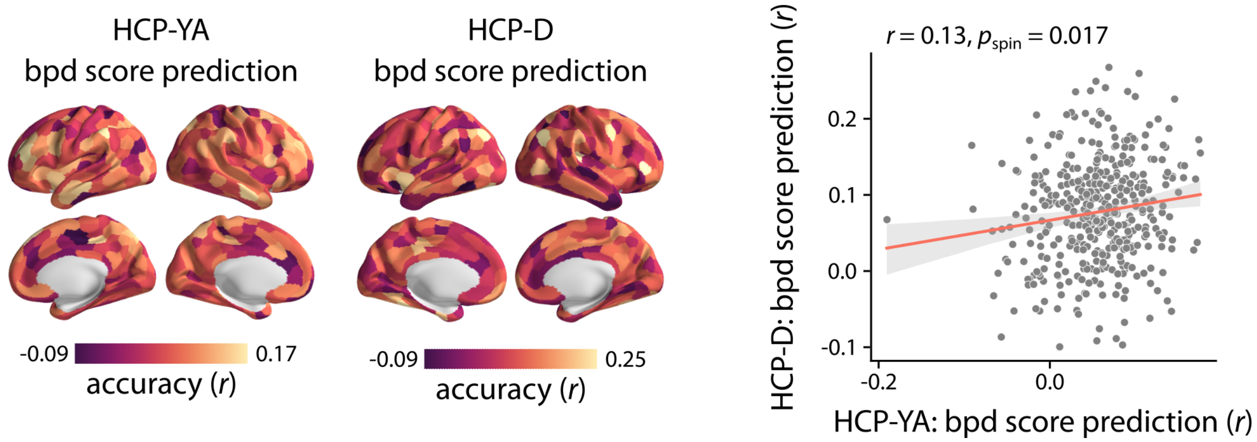

Figure S2. Regional predictive capacity is consistent in adulthood and adolescence | Spatial distributions of BPD score predictions in adulthood (HCP-YA; **Figure 2a**) and adolescence (HCP-D; **Figure 2b**) were compared using Pearson correlation coefficient *r* and 10,000 spatial autocorrelation-preserving null models (i.e., "spin" tests). Each point in the scatter plot corresponds to a brain region.

bioRxiv preprint doi: https://doi.org/10.1101/2023.08.03.551534; this version posted August 21, 2023. The copyright holder for this preprint (which was not certified by peer review) is the author/funder, who has granted bioRxiv a license to display the preprint in perpetuity. It is made available under aCC-BY 4.0 International license.

Table S1. NEO Five Factor Inventory (NEO-FFI) items included in BPD proxy score | The NEO-FFI was used to estimate composite BPD proxy scores (19). The included 24 items and the corresponding questions are listed below. Given that a lower score in items assessing *Agreeableness* and *Conscientiousness* corresponds to more severe BPD symptoms, these items were reverse scored (i.e., multiplied by -1). The composite BPD score was estimated for each participant as the mean score across the included 24 items.

Neo-FFI Factor	Facet	Item Number and Question
Neuroticism (N)	Anxiety	#21: "I often feel tense and jittery."
		#31: "I rarely feel fearful or anxious."
	Angry Hostility	#36: "I often get angry at the way people treat me."
	Depression	#26: "Sometimes I feel completely worthless."
		#41: "Too often when things go wrong, I get discouraged and feel like giving up."
	Vulnerability	#11: "When I'm under a great deal of stress, sometimes I feel like I'm going to pieces."
		#51: "I often feel helpless and want someone else to solve my problems."
Extraversion (E)	Excitement-Seeking	#22: "I like to be where the action is."
Openness (O)	Feelings	#33: "I seldom notice the moods or feelings that different environments produce."
	Actions	#8: "Once I find the right way to do something, I stick to It."
		#28: "I often try new and foreign foods."
Agreeableness (A)	Trust	#24: "I tend to be cynical and skeptical of others' intentions."
		#29: "I believe that most people will take advantage of you if you let them."
	Straightforwardness	#59: "If necessary, I am willing to manipulate people to get what I want."
	Altruism	#14: "Some people think I'm selfish and egotistical."
		#39: "Some people think of me as cold and calculating."
	Compliance	#9: "I often get into arguments with my family and co-workers."
		#54: "If I don't like people, I let them know it."
Conscientiousness (C)	Order	#5: "I keep my belongings clean and neat."
		#55: "I never seem to be able to get organized."
	Dutifulness	#40: "When I make a commitment, I can always be counted on to follow through."
		#45: "Sometimes I'm not as dependable or reliable as I should be."
	Self-Discipline	#30: "I waste a lot of time before settling down to work."
		#50: "I am a productive person who always gets the job done."

bioRxiv preprint doi: https://doi.org/10.1101/2023.08.03.551534; this version posted August 21, 2023. The copyright holder for this preprint (which was not certified by peer review) is the author/funder, who has granted bioRxiv a license to display the preprint in perpetuity. It is made available under aCC-BY 4.0 International license.

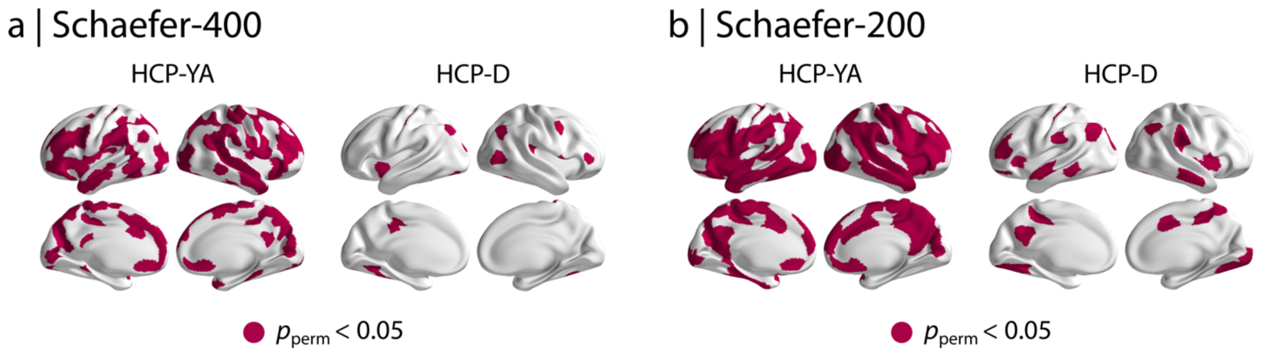

Figure S1. Regional predictive capacity thresholded based on permutation tests | We generated a null distribution of predicted scores for each regional model using 1,000 permutations by randomly shuffling the dependent variable (i.e., BPD scores) and re-calculating the model performance for each permutation. Regional maps were then thresholded based on permutation tests following correction for multiple comparisons ($p_{perm} < 0.05$; FDR-corrected).

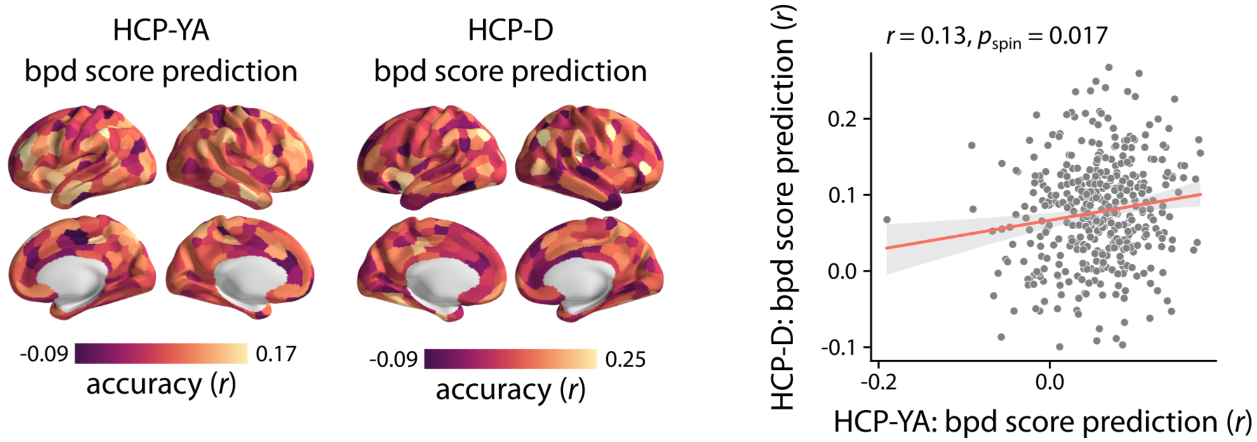

Figure S2. Regional predictive capacity is consistent in adulthood and adolescence | Spatial distributions of BPD score predictions in adulthood (HCP-YA; **Figure 2a**) and adolescence (HCP-D; **Figure 2b**) were compared using Pearson correlation coefficient *r* and 10,000 spatial autocorrelation-preserving null models (i.e., "spin" tests). Each point in the scatter plot corresponds to a brain region.

bioRxiv preprint doi: https://doi.org/10.1101/2023.08.03.551534; this version posted August 21, 2023. The copyright holder for this preprint (which was not certified by peer review) is the author/funder, who has granted bioRxiv a license to display the preprint in perpetuity. It is made available under aCC-BY 4.0 International license.

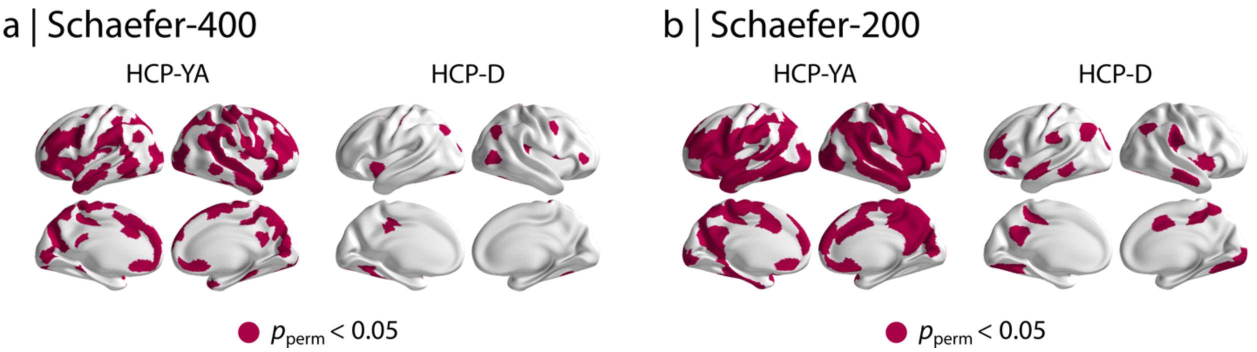

Figure S1. Regional predictive capacity thresholded based on permutation tests | We generated a null distribution of predicted scores for each regional model using 1,000 permutations by randomly shuffling the dependent variable (i.e., BPD scores) and re-calculating the model performance for each permutation. Regional maps were then thresholded based on permutation tests following correction for multiple comparisons ($p_{perm} < 0.05$; FDR-corrected).

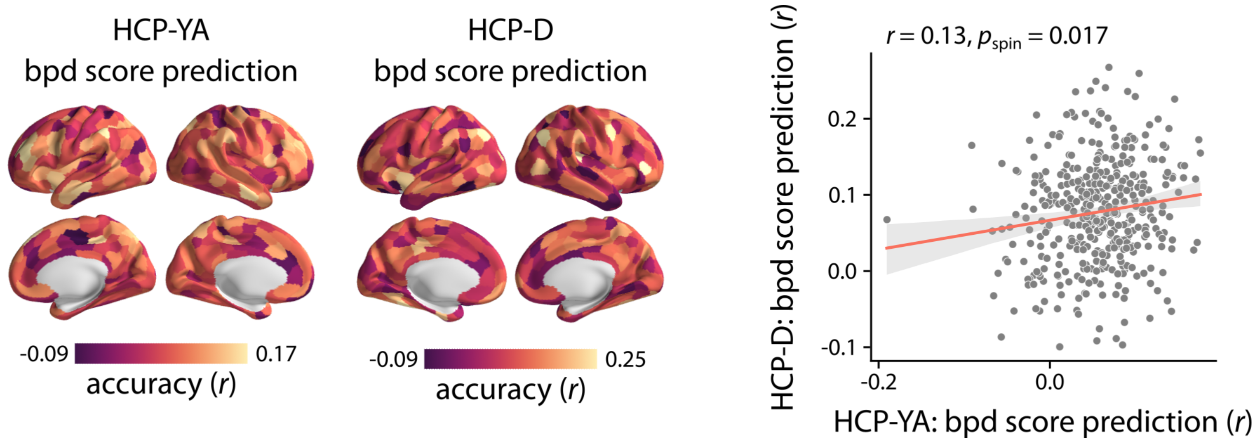

Figure S2. Regional predictive capacity is consistent in adulthood and adolescence | Spatial distributions of BPD score predictions in adulthood (HCP-YA; **Figure 2a**) and adolescence (HCP-D; **Figure 2b**) were compared using Pearson correlation coefficient *r* and 10,000 spatial autocorrelation-preserving null models (i.e., "spin" tests). Each point in the scatter plot corresponds to a brain region.

bioRxiv preprint doi: https://doi.org/10.1101/2023.08.03.551534; this version posted August 21, 2023. The copyright holder for this preprint (which was not certified by peer review) is the author/funder, who has granted bioRxiv a license to display the preprint in perpetuity. It is made available under aCC-BY 4.0 International license.

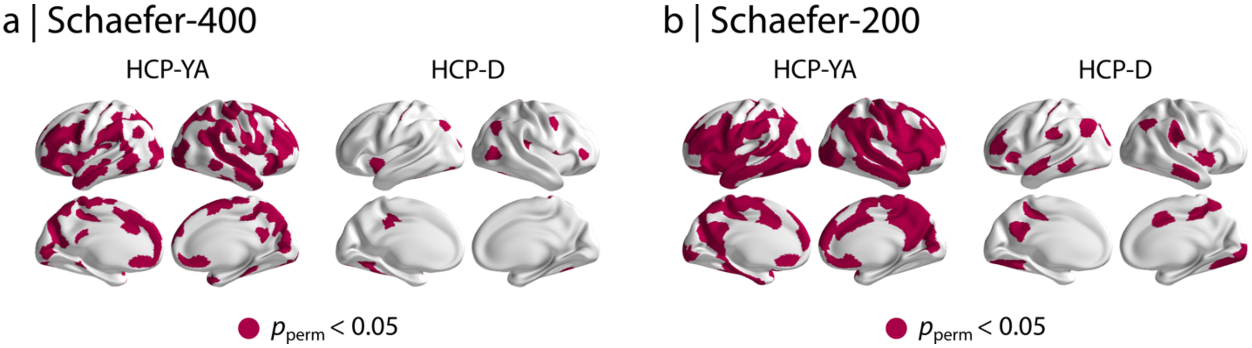

Figure S1. Regional predictive capacity thresholded based on permutation tests | We generated a null distribution of predicted scores for each regional model using 1,000 permutations by randomly shuffling the dependent variable (i.e., BPD scores) and re-calculating the model performance for each permutation. Regional maps were then thresholded based on permutation tests following correction for multiple comparisons (p_{perm} < 0.05; FDR-corrected).

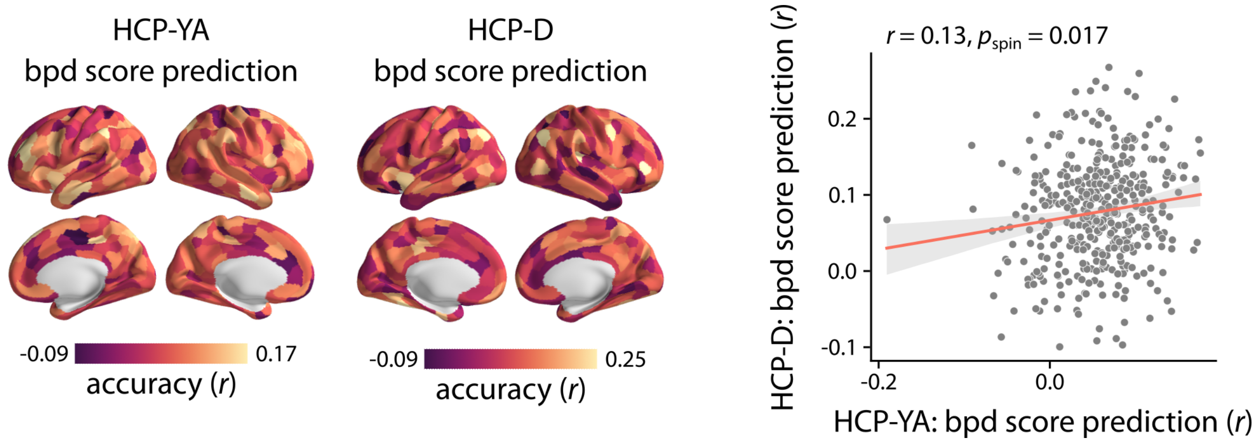

Figure S2. Regional predictive capacity is consistent in adulthood and adolescence | Spatial distributions of BPD score predictions in adulthood (HCP-YA; **Figure 2a**) and adolescence (HCP-D; **Figure 2b**) were compared using Pearson correlation coefficient *r* and 10,000 spatial autocorrelation-preserving null models (i.e., "spin" tests). Each point in the scatter plot corresponds to a brain region.

bioRxiv preprint doi: https://doi.org/10.1101/2023.08.03.551534; this version posted August 21, 2023. The copyright holder for this preprint (which was not certified by peer review) is the author/funder, who has granted bioRxiv a license to display the preprint in perpetuity. It is made available under aCC-BY 4.0 International license.

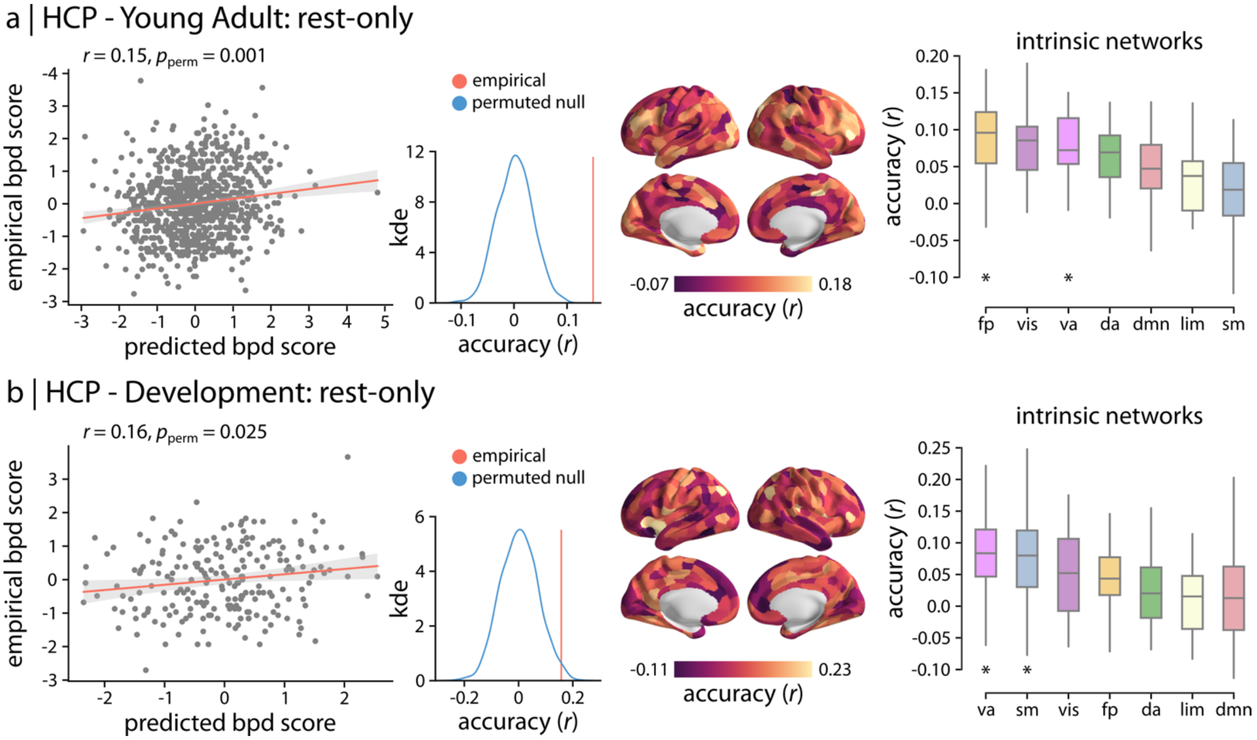

Figure S3. Sensitivity analysis with resting-state fMRI only | Only the resting-state fMRI data were used to predict BPD proxy scores, rather than the concatenated rest- and task-fMRI data used in the original analysis (Figure 2). The results are depicted for (a) healthy young adults from the Human Connectome Project (HCP-YA) data and (b) adolescents from the Human Connectome Project - Development (HCP-D). The participant-level prediction accuracies are shown using the scatter plots (HCP-YA: r = 0.15, 95% CI = [0.08 0.21]; HCP-D: r = 0.16, 95% CI = [0.02 0.28]). Similar to the original analysis, the results were compared with null distributions of accuracies obtained from permutation tests. Region-level accuracies are also shown across the cortex (Schaefer-400 atlas; 99% confidence intervals). Finally, the average functional system-level prediction accuracy was estimated for the 7 intrinsic functional networks. Asterisk denotes significant system-level prediction accuracy based on 10,000 spatial autocorrelation-preserving null models ($p_{spin} < 0.05$; FDR-corrected). Significant system-level accuracy was observed in fronto-parietal (p_{spin} = 0.0001; FDR-corrected) and ventral attention (p_{spin} = 0.014; FDR-corrected) networks for HCP-YA and ventral attention (p_{spin} = 0.0035; FDR-corrected) and somatomotor (p_{spin} = 0.0035; FDR-corrected) networks for HCP-D. Intrinsic networks: vis = visual; sm = somatomotor; da = dorsal attention; va = ventral attention; lim = limbic; fp = fronto-parietal; dmn = default mode.

bioRxiv preprint doi: https://doi.org/10.1101/2023.08.03.551534; this version posted August 21, 2023. The copyright holder for this preprint (which was not certified by peer review) is the author/funder, who has granted bioRxiv a license to display the preprint in perpetuity. It is made available under aCC-BY 4.0 International license.

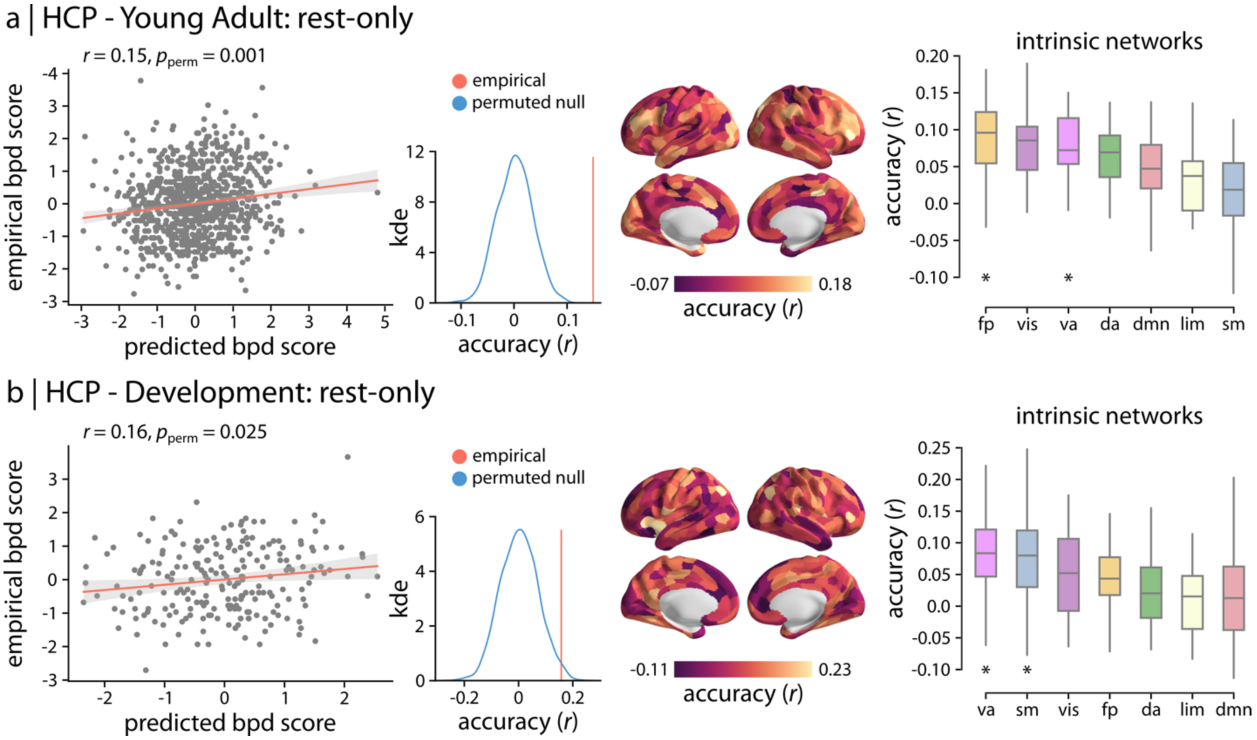

Figure S3. Sensitivity analysis with resting-state fMRI only | Only the resting-state fMRI data were used to predict BPD proxy scores, rather than the concatenated rest- and task-fMRI data used in the original analysis (Figure 2). The results are depicted for (a) healthy young adults from the Human Connectome Project (HCP-YA) data and (b) adolescents from the Human Connectome Project - Development (HCP-D). The participant-level prediction accuracies are shown using the scatter plots (HCP-YA: $r = 0.15$, 95% CI = [0.08 0.21]; HCP-D: $r = 0.16$, 95% CI = [0.02 0.28]). Similar to the original analysis, the results were compared with null distributions of accuracies obtained from permutation tests. Region-level accuracies are also shown across the cortex (Schaefer-400 atlas; 99% confidence intervals). Finally, the average functional system-level prediction accuracy was estimated for the 7 intrinsic functional networks. Asterisk denotes significant system-level prediction accuracy based on 10,000 spatial autocorrelation-preserving null models ($p_{spin} < 0.05$; FDR-corrected). Significant system-level accuracy was observed in fronto-parietal ($p_{spin} = 0.0001$; FDR-corrected) and ventral attention ($p_{spin} = 0.014$; FDR-corrected) networks for HCP-YA and ventral attention ($p_{spin} = 0.0035$; FDR-corrected) and somatomotor ($p_{spin} = 0.0035$; FDR-corrected) networks for HCP-D. Intrinsic networks: vis = visual; sm = somatomotor; da = dorsal attention; va = ventral attention; lim = limbic; fp = fronto-parietal; dmn = default mode.

bioRxiv preprint doi: https://doi.org/10.1101/2023.08.03.551534; this version posted August 21, 2023. The copyright holder for this preprint (which was not certified by peer review) is the author/funder, who has granted bioRxiv a license to display the preprint in perpetuity. It is made available under aCC-BY 4.0 International license.

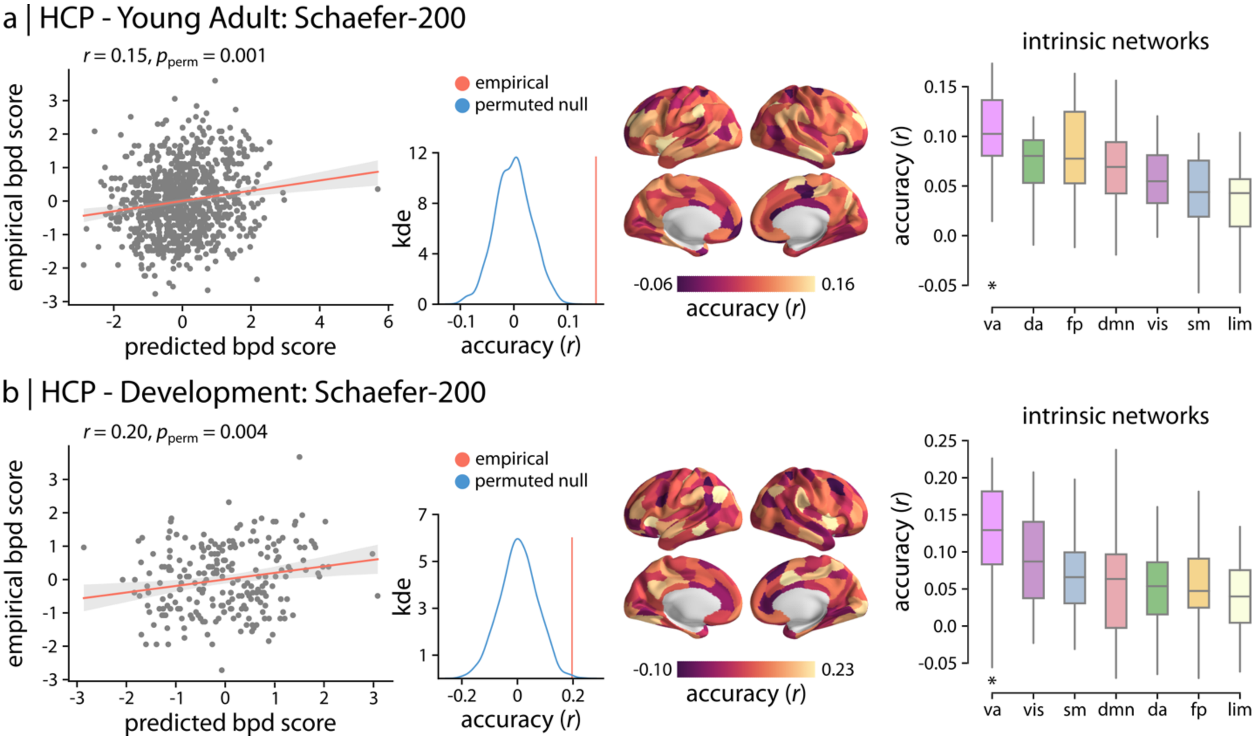

Figure S4. Sensitivity analysis with lower parcellation resolution | To ensure that the findings are independent from the parcellation resolution, a lower resolution atlas with 200 cortical regions (Schaefer-200 atlas) was used to obtain functional connectivity matrices. Functional connectivity data were then used to predict BPD proxy scores. The results are depicted for (a) healthy young adults from the Human Connectome Project (HCP-YA) data and (b) adolescents from the Human Connectome Project - Development (HCP-D). The participant-level prediction accuracies are shown using the scatter plots (HCP-YA: r = 0.15, 95% CI = [0.09 0.22]; HCP-D: r = 0.20, 95% CI = [0.06 0.32]). Similar to the original analysis, the results were compared with null distributions of accuracies obtained from permutation tests. Region-level accuracies are also depicted across the cortex (Schaefer-400 atlas; 99% confidence intervals). Finally, the average functional system-level prediction accuracy was estimated for the 7 intrinsic functional networks. Asterisk denotes significant system-level prediction accuracy based on 10,000 spatial autocorrelation-preserving null models ($p_{spin} < 0.05$; FDR-corrected). Significant system-level accuracy was observed in ventral attention network for HCP-YA (p_{spin} = 0.0001; FDR-corrected) and HCP-D (p_{spin} = 0.0001; FDR-corrected). Intrinsic networks: vis = visual; sm = somatomotor; da = dorsal attention; va = ventral attention; lim = limbic; fp = fronto-parietal; dmn = default mode.

bioRxiv preprint doi: https://doi.org/10.1101/2023.08.03.551534; this version posted August 21, 2023. The copyright holder for this preprint (which was not certified by peer review) is the author/funder, who has granted bioRxiv a license to display the preprint in perpetuity. It is made available under aCC-BY 4.0 International license.

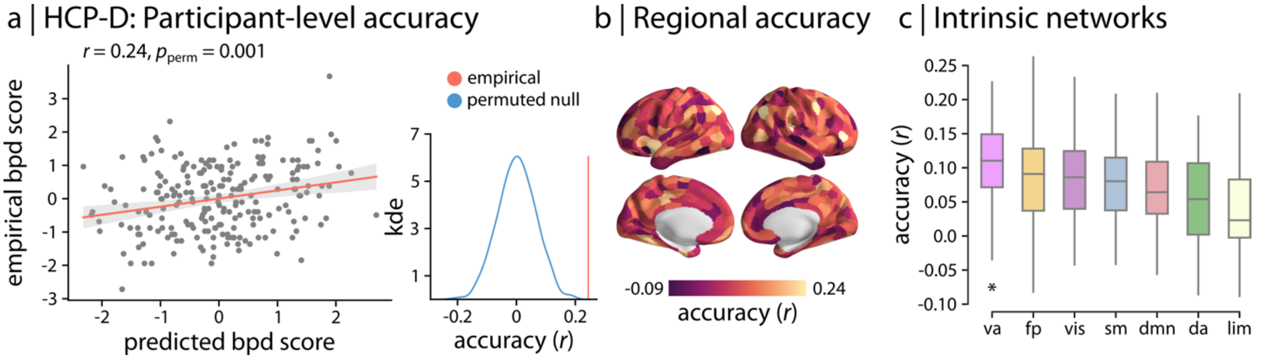

Figure S5. Findings are not influenced by scanning-site effects | We used CovBat-GAM to harmonize functional connectivity across scanning sites in HCP-D and repeated the analyses with harmonized data. Participant-level (a), region-level (b), and system-level (c) accuracies were consistent with the original results. Asterisk denotes significant system-level prediction accuracy (p_{spin} < 0.05; FDR-corrected). Significant system-level accuracy was observed in ventral attention network (p_{spin} = 0.0001; FDR-corrected). Intrinsic networks: vis = visual; sm = somatomotor; da = dorsal attention; va = ventral attention; lim = limbic; fp = fronto-parietal; dmn = default mode.
